# Supplementary material for: Down-Regulation of HLA-C Expression on Melanocytes May Contribute to the Therapeutic Efficacy of UVB Phototherapy in Psoriasis
Source: Int J Mol Sci. 2025 Mar 21;26(7):2858. doi: 10.3390/ijms26072858 (PMC11988605; doi:10.3390/ijms26072858)
Supplement: Supplementary file 1 [file ijms-26-02858-s001.zip › ijms-3450506-supplementary.pdf]

Down-Regulation of HLA-C Expression on Melanocytes May Contribute to the Therapeutic Efficacy of UVB Phototherapy in Psoriasis

Authors: Yukiyasu Arakawa, Akiko Arakawa, Secil Vural, Mengwen He, Sigrid Vollmer, Jörg C. Prinz

SUPPLEMENTAL FIGURES S1-S7

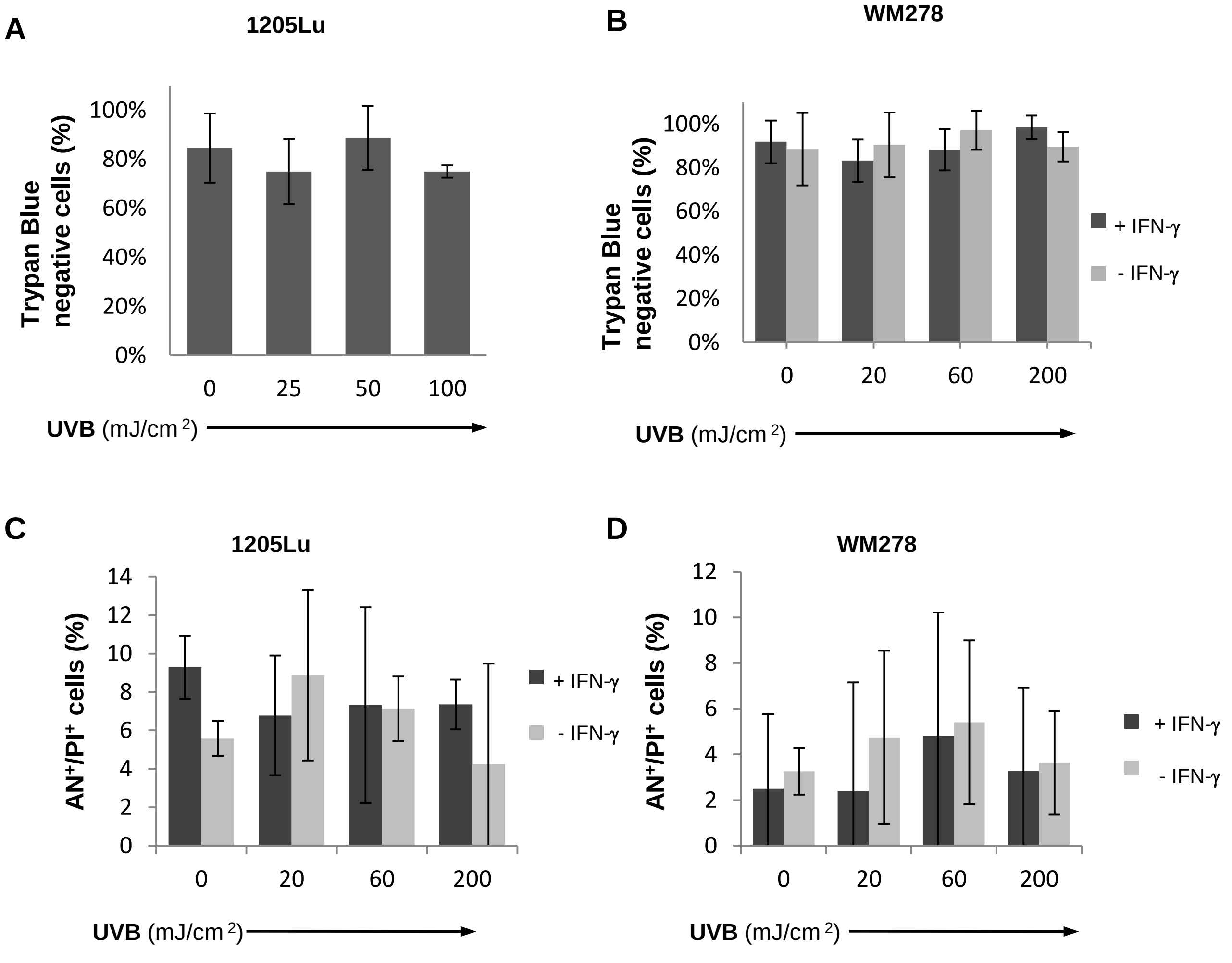

Figure S1. **The applied UVB doses hardly affected melanocyte viability.** Percentage of viable (**A**) 1205Lu or (**B**) WM278 cells determined by Trypan blue staining or apoptotic (**C**). 1205Lu or (**D**). WM278 cells as determined by Cy™5 Annexin V binding and 10µg/ml propidium iodide staining 24 hrs after irradiation with escalating doses of UVB with or without IFN-γ preincubation. In (**A**) and (**B**), stained cells were determined in an inverted microscope, in (**C**) and (**D**) measured by flow cytometry and data analyzed using Flow Jo software. Data are given as mean ± s.e.m. of each three independent experiments.

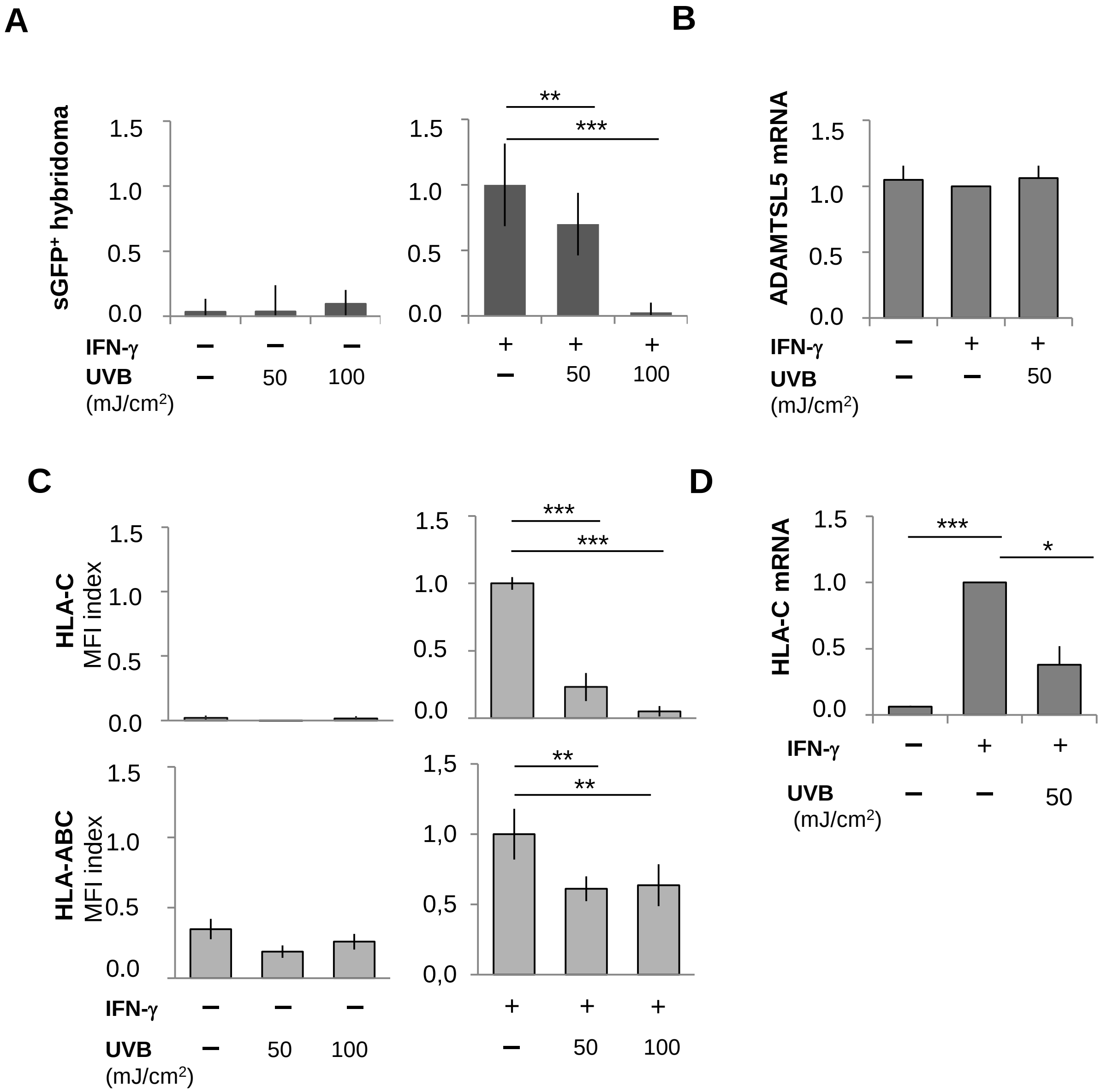

Figure S2. **Effect of IFN-γ and UVB on Vα3S1/Vβ13S1-TCR hybridoma stimulation, HLA-class I and ADAMTSL5 expression by HLA-C*06:02⁺ 1205Lu cells**. (**A**) Vα3S1/Vβ13S1-TCR hybridoma activation by co-culture with 1205Lu cells grown without or with IFN-γ and irradiated with 50 or 100 mJ/cm^2 UVB. (**B**) Effect of IFN-γ and UVB irradiation on relative ADAMTSL5 transcript levels in 1205Lu cells as assessed by qPCR. (**C**) Effect of UVB-irradiation on spontaneous or IFN-γ-induced expression of HLA-C or HLA-ABC. Mean fluorescence intensity (MFI) of HLA-C (DT9) or HLA-ABC (W6/32) is normalized to non-UVB plus IFN-γ MFI data of each analysis. (**D**) Effect of IFN-γ and UVB irradiation on relative HLA-C transcript levels in 1205Lu cells assessed by qPCR. All data summarize technical triplicates from three or more independent experiments shown as mean ± s.e.m. and compared by Mann-Whitney U-test (*, $p < 0.05$, **, $p < 0.01$, ***, $p < 0.005$). In qPCR experiments, water served as negative control.

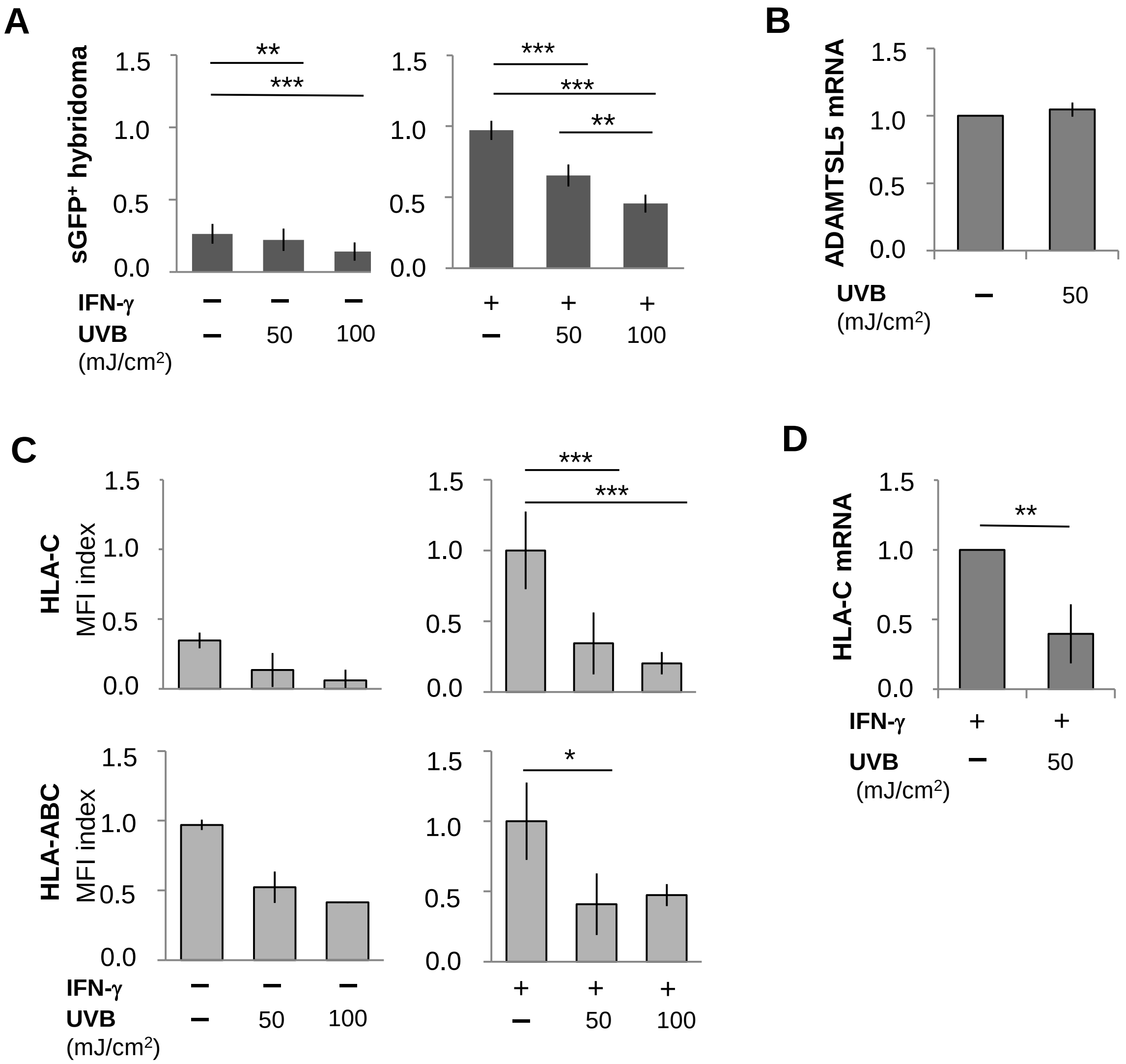

Figure S3. Effect of IFN-γ and UVB on Vα3S1/Vβ13S1-TCR hybridoma stimulation, HLA-class I and ADAMTSL5 expression by HLA-C*06:02⁺ WM793 cells. (**A**) Vα3S1/Vβ13S1-TCR hybridoma activation by co-culture with WM793 cells grown with or without IFN-γ and irradiated with 50 or 100 mJ/cm^2. (**B**) Effect of UVB irradiation on relative ADAMTSL5 transcript levels in WM793 cells as assessed by qPCR. (**C**) Effect of UVB-irradiation on the spontaneous or IFN-γ-induced expression of HLA-C or HLA-ABC. MFI of HLA-C (DT9) and HLA-ABC (W6/32) of WM793 cells is normalized to non-UVB plus IFN-γ MFI data of each analysis. (**D**) Effect of UVB irradiation on the IFN-γ-induced HLA-C transcript levels as assessed by qPCR. In qPCR experiments, water served as negative control. All data summarize technical triplicates from three or more independent experiments shown as mean ± s.e.m. and compared by Mann-Whitney U-test (*, $p < 0.05$, **, $p < 0.01$, ***, $p < 0.005$).

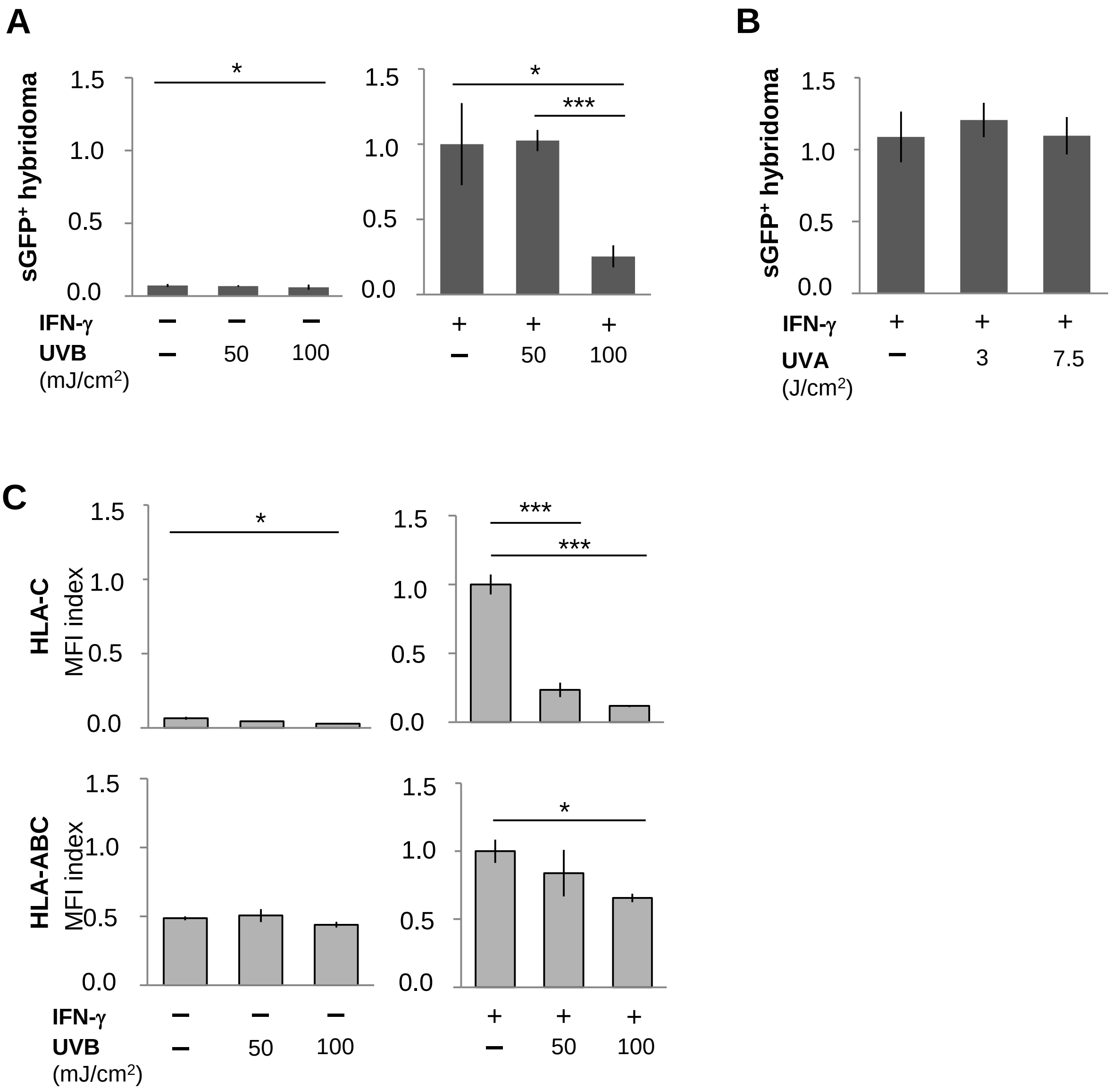

Figure S4. Irradiation of HLA-C*06:02+ WM278 cells with UVB but not UVA decreases activation of the autoreactive psoriatic Vα3S1/Vβ13S1 TCR. Vα3S1/Vβ13S1-TCR hybridoma stimulation by co-culture with WM278 cells grown without or with IFN-γ and irradiated with 50 or 100 mJ/cm² UVB (**A**) or 3 or 7.5 mJ/cm² UVA (**B**). (**C**) Effect of UVB-irradiation on the spontaneous or IFN-γ-induced expression of HLA-C or HLA-ABC. MFI of HLA-C (DT9) and HLA-ABC (W6/32) of WM278 cells is normalized to non-UVB plus IFN-γ MFI data of each analysis. Data summarizing technical triplicates from three or more independent experiments are shown as mean ± s.e.m. and compared by Mann-Whitney U-test (*, $p < 0.05$, ***, $p < 0.005$).

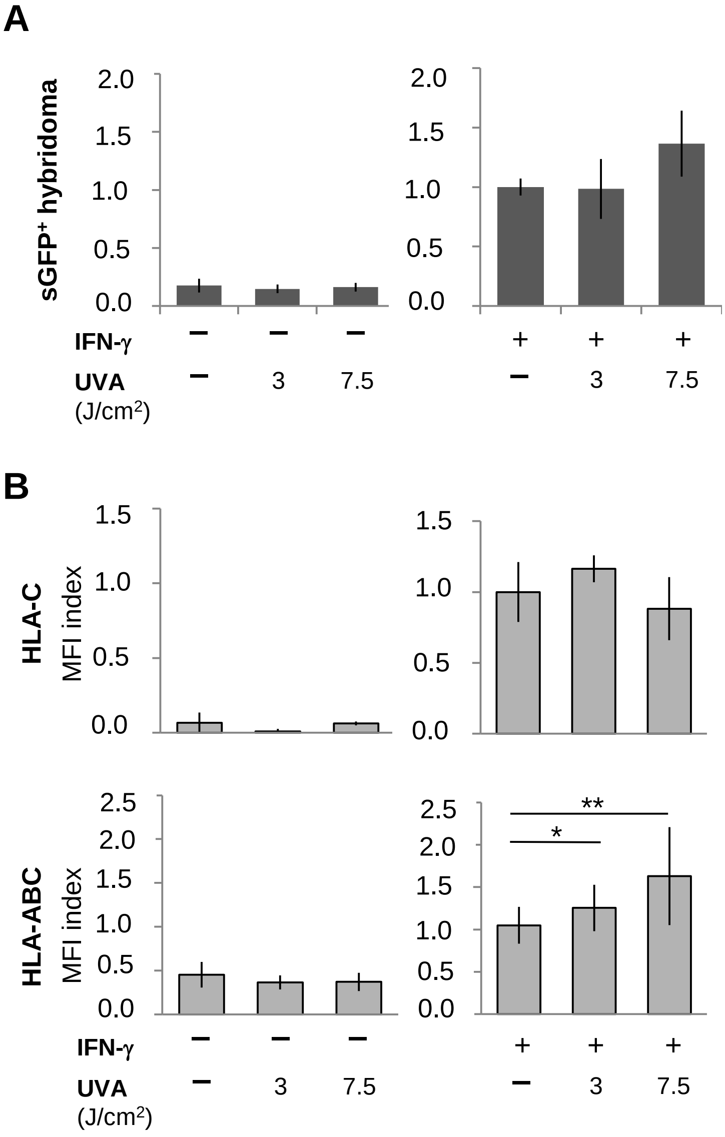

Figure S5. Effect of UVA irradiation of 1205Lu cells on Vα3S1/Vβ13S1-TCR hybridoma stimulation and HLA-class I expression. (**A**) Effect of UVA irradiation of 1205Lu cells grown without or with IFN-γ on Vα3S1/Vβ13S1-TCR hybridoma stimulation. (**B**) Effect of UVA-irradiation on the spontaneous or IFN-γ-induced expression of HLA-C or HLA-ABC. MFI of HLA-C (DT9) and HLA-ABC (W6/32) of 1205Lu cells is normalized to the MFI of non-irradiated cells plus IFN-γ of each analysis. Data summarizing technical triplicates from three or more independent experiments are shown as mean ± s.e.m. and compared by Mann-Whitney U-test (*, $p < 0.05$, **, $p < 0.05$).

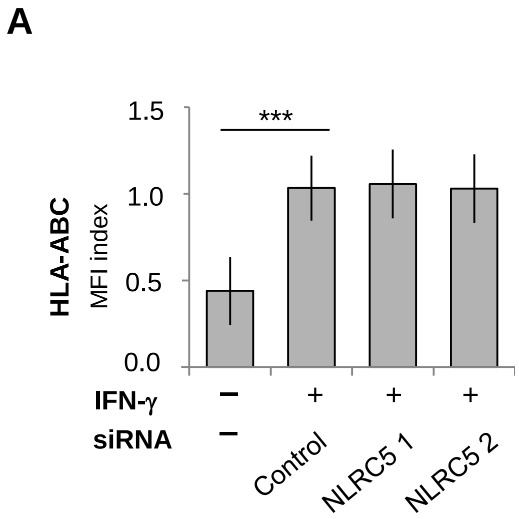

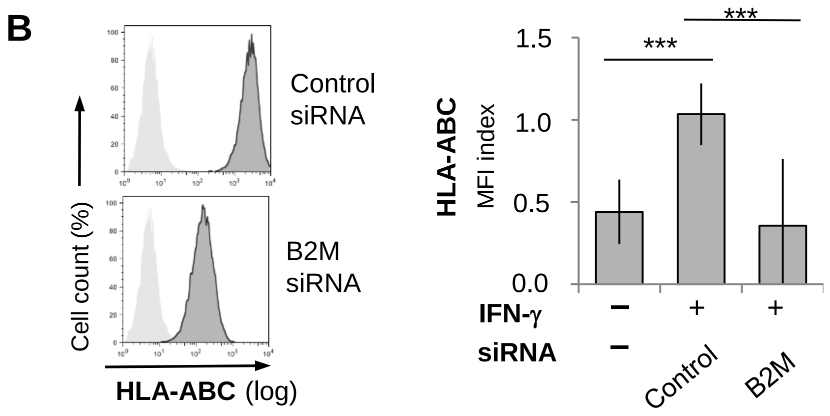

Figure S6. Effect of knockdown of NLRC5 or B2M on the expression of HLA-ABC. Expression of HLA-ABC following knockdown of (**A**) NLRC5 or (**B**) B2M in 1205LU cells without or with preincubation with IFN-γ was determined by staining with W6/32 staining and flow cytometry analysis. Histograms in B show the reduction of IFN-γ-induced HLA-ABC expression by B2M siRNA. Data summarizing technical triplicates from three or more independent experiments are shown as mean ± s.e.m. and compared by non-paired t-test (*, $p < 0.05$, ***, $p < 0.005$).

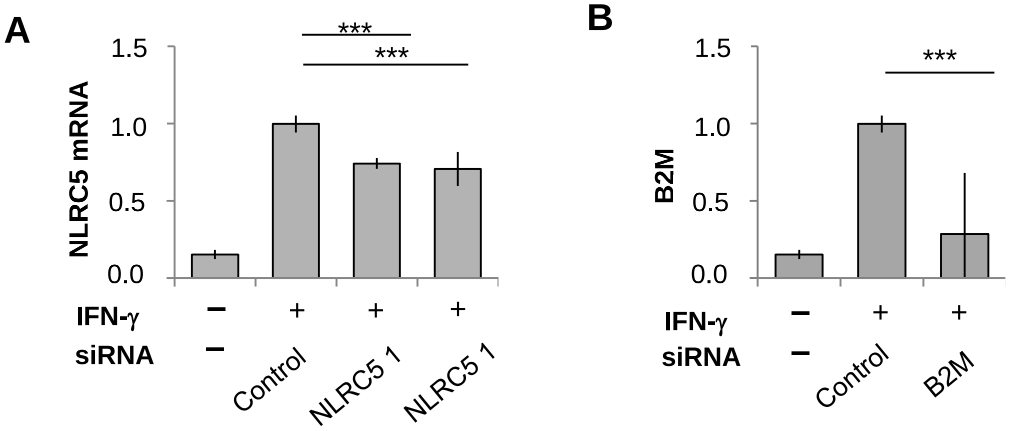

Figure S7. Validation of Knockdown of (**A**) NLRC5 or (**B**) B2M as determined by qPCR. Data are normalized to control plus IFN-γ.
